# Radiation-Free Therapy for the Initial Treatment of Good Prognosis Early Non-Bulky Hodgkin Lymphoma, Defined by a Low Metabolic Tumor Volume and a Negative Interim PET After 2 Chemotherapy Cycles: The RAFTING Trial Protocol

**DOI:** 10.3390/biomedicines14081861

**Published:** 2026-08-19

**Authors:** Kateryna Filonenko, Marco Picardi, Stephane Chauvie, Andrea Riccardo Filippi, Maria Cristina Pirosa, Luca Guerra, Federico Fallanca, Marta Bednarek, Michał Kurlapski, Eva Domingo-Domenech, Andrea Visentin, Caterina Patti, Ramón García-Sanz, Javier Nunez, Javier Lopez-Jiménez, Agnieszka Giza, Adam Wyszomirski, Alessandro Rambaldi, Davide Rossi, Anna Sureda, Andrea Gallamini, Jan Maciej Zaucha

**Affiliations:** 1 Department of Hematology, Transplantology, and Cell Therapies, University Medical Center in Gdansk, 80-214 Gdansk, Poland; ksfilonenko@yahoo.com (K.F.);; 2 Department of Clinical Medicine and Surgery, AOU Federico II, 80131 Naples, Italy; 3 Medical Physics Division, Santa Croce e Carle General Hospital, 12100 Cuneo, Italy; 4 Radiation Oncology, Fondazione IRCCS Istituto Nazionale dei Tumori and Department of Oncology, University of Milan, 20133 Milan, Italy; andreariccardo.filippi@istitutotumori.mi.it; 5 Laboratory of Experimental Hematology, Institute of Oncology Research, 6500 Bellinzona, Switzerland; 6 School of Medicine and Surgery, University of Milano Bicocca, 20126 Milan, Italy; 7 Nuclear Medicine Unit, Fondazione IRCCS San Gerardo dei Tintori, 20900 Monza, Italy; 8 IRCCS San Raffaele Scientific Institute, Nuclear Medicine, 20132 Milan, Italy; 9 2nd Division of Radiology, Medical University of Gdansk, 80-214 Gdansk, Poland; 10 Department of Hematology, Transplantology, and Cell Therapies, Medical University of Gdansk, 80-210 Gdansk, Poland; 11 Hematology Department, Institut Catala d’Oncologia, 08908 Barcelona, Spain; 12 Hospital Duran i Reynals, 08908 Barcelona, Spain; 13 The Bellvitge Biomedical Research Institute, 08908 Barcelona, Spain; 14 Hematology Unit, Department of Medicine (DIMED), University of Padua in Padua, 35128 Padua, Italy; 15 U.O.C. di Oncoematologia, Ospedali Riuniti Villa Sofia—Cervello (Presidio V. Cervello), 90146 Palermo, Italy; 16 Hematology Division, Hospital Universitario de Salamanca, 37007 Salamanca, Spain; 17 Hematology Service, Hospital Universitario Marqués de Valdecilla (HUMV), 39008 Santander, Spain; 18 Hematology and Chemotherapy Department, Hospital Universitario Ramón y Cajal Madrid, IRYCIS, Universidad Alcalá de Henares, 28034 Madrid, Spain; 19 Department of Haematology, Jagiellonian University Medical College, 30-688 Krakow, Poland; 20 Department of Adult Neurology, Faculty of Medicine, Medical University of Gdansk, 80-214 Gdansk, Poland; 21 Hematology and Oncology Department, ASST Papa Giovanni XXIII Hospital in Bergamo, 24127 Bergamo, Italy; 22 Research and Clinical Innovation Department, Lacassagne Cancer Center, 06189 Nice, France

**Keywords:** early-stage Hodgkin’s lymphoma, risk stratification, individualized treatment, radiation-free treatment, cfDNA, total metabolic tumor volume, nivolumab, combined modality treatment

## Abstract

Radiation-free treatment for early-stage classic Hodgkin lymphoma (eHL) has been shown to be less effective than standard combined-modality treatment (CMT; chemotherapy plus involved-node radiotherapy (INRT)), which achieves long-term disease control of 94–95%. Approximately 70% of patients can be cured with chemotherapy alone, whereas about 5% fail CMT. Identifying patients who can safely receive chemotherapy alone and those requiring intensified CMT could enable a risk-adapted treatment strategy. The RAFTING trial (NCT04866654; EudraCT 2020-002382-33) is an international, prospective, phase 2, non-inferiority study enrolling patients 18–70 years, stage I–IIA eHL without bulky disease, B symptoms, or extranodal involvement. Low-risk (LR) patients are defined by total metabolic tumor volume (TMTV) <84 mL and negative PET-2. Those with at least one modified EORTC (mEORTC) risk factor, in which bulky disease is replaced by a large nodal mass (5–10 cm), receive four ABVD cycles, while those without risk factors receive two ABVD cycles alone. High-risk (HR) patients, defined by TMTV ≥84 mL and/or positive PET-2, receive “triple therapy”: 4 ABVD cycles, INRT (20/30 Gy), and nivolumab (240 mg q2w, ≤doses). LR patients are monitored using cfDNA. Limited relapse is treated with INRT (36 Gy) and nivolumab. The RAFTING trial is the first prospective eHL study to personalize treatment using TMTV and PET-2. It aims to omit radiotherapy in LR patients, intensify treatment in HR patients, and spare relapsed LR patients high-dose chemotherapy and autologous transplantation. CfDNA is being evaluated as a relapse marker. Despite the protocol’s complexity, this study exemplifies personalized medicine and could transform treatment practices.

## 1. Introduction

Patients with early-stage Hodgkin lymphoma (eHL) have a heterogeneous prognosis: approximately 60–70% can be cured with ABVD chemotherapy (doxorubicin, bleomycin, vinblastine, dacarbazine) (chemotherapy) alone, while 20–30% require combined modality treatment (CMT) with chemotherapy followed by involved-node radiotherapy (INRT). Despite an excellent treatment outcome after CMT, 5–10% of patients fail to achieve a cure due to disease progression or relapse [1]. Despite a high disease-specific survival rate (90–95%), the eHL treatment remains associated with a definite long-term treatment-related morbidity and mortality (TRM), resulting in impaired overall survival (OS) compared to an age- and sex-matched general population [2]. TRM includes complications related to both chemotherapy and radiotherapy (RT). Late effects, such as cardiovascular complications and secondary malignancies, are particularly associated with RT [3]. Despite a reduction of RT doses to 30 and 20 Gy, the use of modern conformal techniques, intensity-modulated RT, and precise 3D imaging for clinical target volume delineation, the risk of late RT-induced toxicity is still a concern [4]. Moreover, the TRM related to high-dose chemotherapy (HDCT) and autologous hematopoietic cell transplantation (auto-HCT) for disease rescue additionally affects long-term toxicity and survival of relapsed patients [5].

Previous studies of radiation-free treatment (RFT) in stage I–IIA HL patients with classic Hodgkin lymphoma without B symptoms and extra-nodal disease (eHL), the RAPID, H10, HD16 clinical trials, failed to demonstrate non-inferiority within margins of 7–10% [6,7,8]. However, in those trials, approximately 60–70% of patients were cured with chemotherapy alone. On the other hand, a small but non-negligible percentage of patients ultimately experience disease relapse after CMT. Therefore, an unmet need exists for a better stratification of eHL to tailor therapy according to individual disease risk, with the goal of preserving efficacy while minimizing RT-related TRM and with a better prognostic patient stratification to single out the small patient subset failing CMT. In these studies, cure with chemotherapy alone was achieved mainly in patients with negative interim positron emission tomography (PET) after the second chemotherapy cycle (PET-2). Conversely, approximately 25% of patients with a positive PET-2 (H10 trial) did not respond to standard CMT [7]. To date, PET-2 is regarded the strongest predictor of treatment failure not only in advanced disease but also in early-stage disease [7]. Classical bulky disease is a well-known unfavorable prognostic factor. Consistently, in a retrospective reappraisal of predictive factors for treatment outcome in the RAPID trial, a large nodal mass with the largest diameter ≥ 5 cm (LNM), measured on baseline contrast-enhanced computed tomography (CT), emerged as a strong predictor of treatment failure (HR 3.78, 95% CI 1.59–9.02; *p* = 0.003) [9]. The total metabolic tumor volume (TMTV) calculated on the PET/CT performed at baseline also proved to be of prognostic value in eHL, although different methods for TMTV computing have been proposed and different cut-off values have been adopted [10]. In patients enrolled in the standard arm of the H10 trial, a TMTV value > 147 mL indicated a risk of treatment failure 21% higher compared to that of patients with a lower value. [5]. However, in 129 non-bulky stage I–IIA patients from the French cohort of the H10 study, who matched the inclusion criteria of the RAFTING trial, a much lower value of 84 mL proved to be the most accurate threshold for predicting favorable treatment outcome. Patients with a TMTV value > 84 mL had a treatment failure rate of 44%, compared with 4% among patients with a lower value (unpublished observation, Meignan M, personal communication, 2019). Finally, the H10 study showed that about 12.6% of RFT patients relapsed, and among them, 73% exclusively in the initially involved regions [7].

On the other hand, no data exist on the efficacy of RT rescue after first-line chemotherapy treatment alone. One of the older studies (from the 1990s) showed a 10-year disease-free survival in 30% of patients failing first-line chemotherapy and rescued with extended field radiotherapy [11].

Thirty years later, the monoclonal antibodies directed against the PD-1 immune checkpoint on T lymphocytes have demonstrated strong efficacy in both relapsed/refractory and untreated classic HL. When administered during or after INRT, anti-PD1 blockade may improve INRT efficacy through an abscopal effect and enhance a durable long-term disease control in high-risk patients [12,13].

Based on these data, the RAFTING trial was designed to verify a risk-adapted therapeutic strategy for stratifying eHL patients with favorable (I–IIA nonbulky) disease into low-risk (LR) patients who could be safely treated with chemotherapy alone, without consolidation radiotherapy, and high-risk (HR) patients, who would benefit from standard CMT followed by the anti-PD1 monoclonal antibody nivolumab. The study was also aimed to determine whether the association of RT and nivolumab could rescue LR patients failing chemotherapy with the criteria of “limited relapse” (see below). Thus, the study intends to preserve a high efficacy of the proposed overall therapeutic strategy, while reducing treatment-related toxicity and long-term morbidity.

## 2. Methods and Analysis

### 2.1. Study Design

The RAFTING trial (NCT04866654, EudraCT 2020-002382-33) is a prospective, multicenter, international (Poland, Italy, Spain), single-arm, non-inferiority, phase 2 study.

### 2.2. Enrolled Population

Untreated, favorable stage I–IIA with histologically confirmed classical eHL according to the current World Health Organization (WHO) classification, aged 18–70, without classical bulky (>10 cm), B symptoms, or extranodal disease are eligible for study enrollment. Other inclusion/exclusion criteria are standard for prospective onco-hematological studies.

### 2.3. Patient Stratification and Treatment

Stratification is performed in two steps to define the low- and high-risk categories.

**Figure 1:**
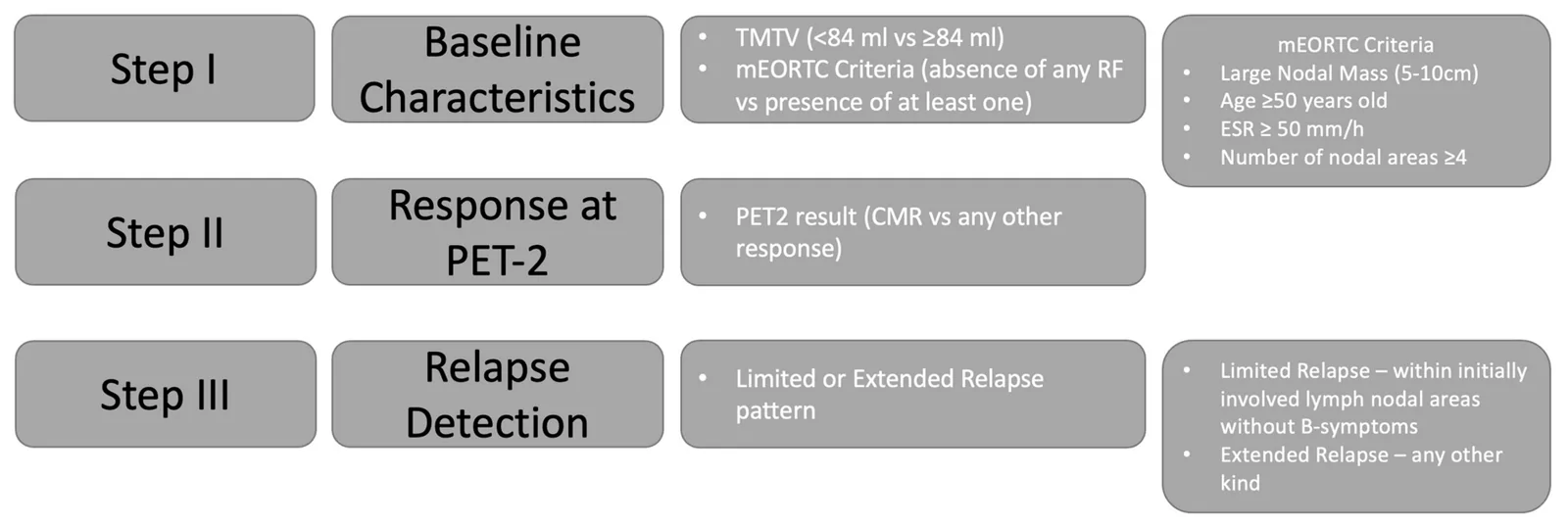
Risk factors and stratification steps. CMR—complete metabolic response, ESR—erythrocyte sedimentation rate, mEORTC—modified EORTC criteria, PET-2—positron emission tomography after second chemotherapy cycle, TMTV—total metabolic tumor volume.

The first is based on the TMTV value at baseline (low or high) and presence/absence of modified EORTC (mEORTC) criteria, in which the classical bulky disease (defined as a nodal mass larger than 10 cm) is replaced by a “large nodal mass” (LNM) of 5–10 cm in diameter (Figure 1). The second step is after the second chemotherapy cycle and is based on PET-2 results.

**Figure 2:**
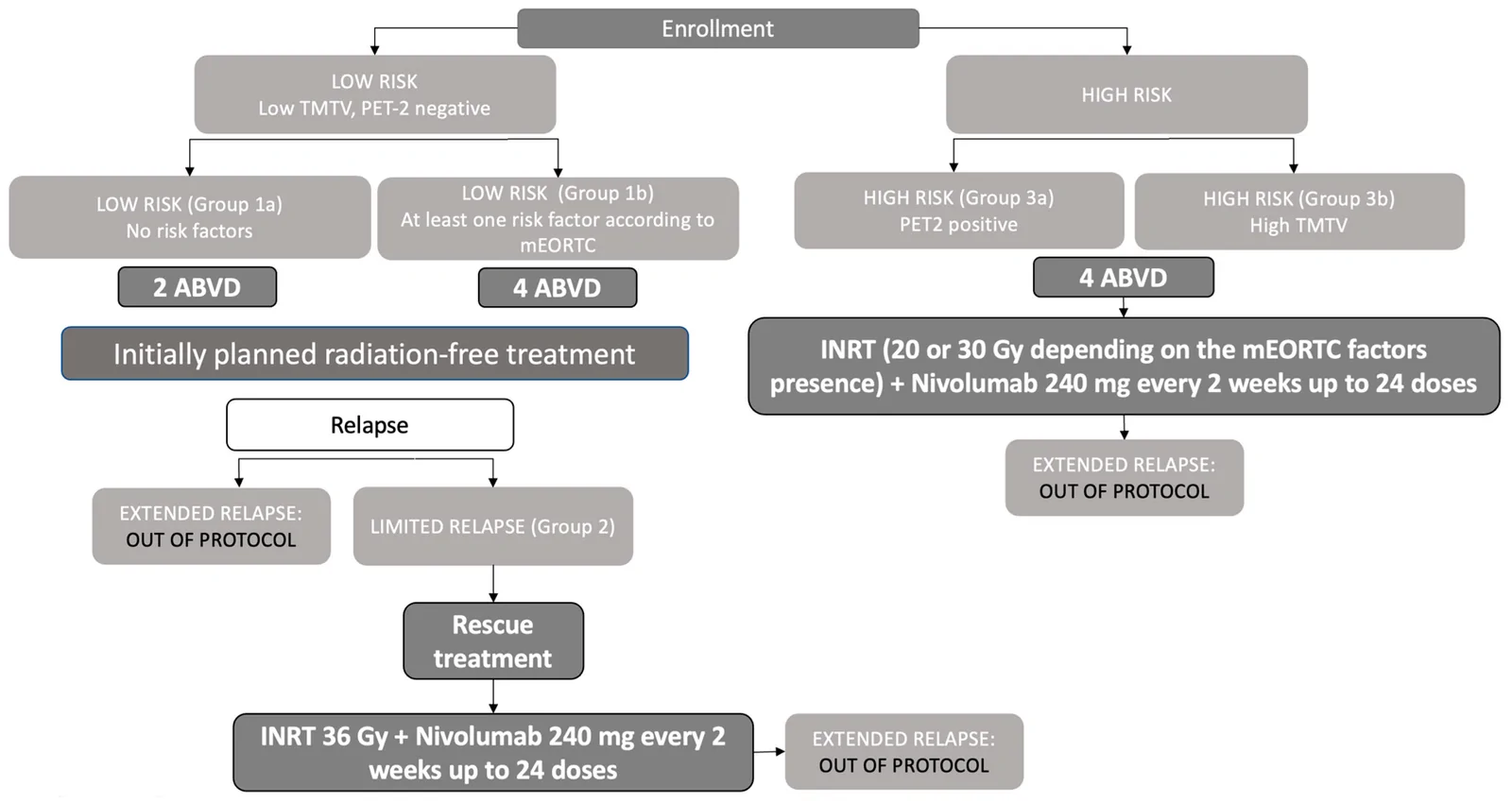
Study treatment scheme. ABVD—doxorubicin, bleomycin, vinblastine, dacarbazine, Gy—Gray, INRT—involved node radiation therapy, mEORTC—modified EORTC criteria, PET-2—positron emission tomography after second chemotherapy cycle, TMTV—total metabolic tumor volume.

The low-risk (LR) group is defined by a baseline TMTV < 84 mL and negative interim PET-2 (Deauville scale; DS: 1–3). Within this category, patients are further split into two more categories: group 1a—patients without mEORTC risk factors that receive two chemotherapy cycles only, and group 1b—patients with at least one risk factor according to the mEORTC criteria that receive four chemotherapy cycles (Figure 2). The ABVD chemotherapy includes doxorubicin 25 mg/m^2^, bleomycin 10 IU/m^2^, vinblastine 6 mg/m^2^, and dacarbazine 375 mg/m^2^, administered intravenously on days 1 and 15 of each 28-day cycle.

The HR group includes patients with either a positive PET-2 (DS 4–5, group 3a) and/or baseline TMTV ≥ 84 mL (group 3b). Both patient subsets receive a “triple therapy,” consisting of four chemotherapy cycles, INRT at either 20 or 30 Gy depending on the absence or presence of baseline mEORTC risk factors, respectively, followed by Nivolumab maintenance administered every two weeks at a dose of 240 mg intravenously over 30 min for up to one year, for a maximum of 24 doses (Figure 2). This duration was selected based on the CheckMate 205 and KEYNOTE-087 studies [12,13]. The newly diagnosed advanced-stage HL patients (CheckMate 205 study, group D) received up to 16 doses of nivolumab (4 doses of monotherapy and 12 in combination with AVD chemotherapy). The relapsed/refractory patients received nivolumab up to disease progression or unacceptable toxicity, with a possibility of treatment discontinuation in group C for patients with CR for 1 year. In both cases, acceptable CR rate and duration were achieved. The KEYNOTE-087 data show that 1 year of PD-1 blockade is associated with sustained remissions [14]. Recognizing that the optimal duration of anti-PD-1 therapy in this setting remains unknown, this study will explore correlations between treatment duration, toxicity, and outcomes to inform future trials.

In the LR group, two different patterns of disease relapse are defined (limited and extended relapse). Limited relapse is characterized by nodal recurrence in the initially involved regions, with or without a maximum of three new nodal sites, and absence of B symptoms, which allows the use of INRT as rescue and does not exceed the field of radiation therapy. The consistency of each relapse with the predefined criteria of limited relapse was assessed by a panel of PET reviewers and a medical monitor. Limited relapses are treated with INRT at 36 Gy followed by nivolumab according to the same schedule applied in the HR groups (group 2).

Patients requiring RT according to the study protocol received INRT within 4–6 weeks after completion of chemotherapy. The RT techniques and safety constraints followed the guidelines of the International Lymphoma Radiation Oncology Group and Fondazione Italiana Linfomi Radiotherapy Committee [15,16].

INRT was delivered using conventional fractionation (2 Gy per fraction, five fractions per week). The prescribed dose was 20 Gy in 10 fractions for patients with early favorable disease and 30 Gy in 15 fractions for patients with early unfavorable disease. Patients experiencing a limited nodal relapse after chemotherapy alone could receive salvage INRT to the initially involved nodal sites and up to three additional nodal regions, with a total dose of 36 Gy in 18 fractions.

Target volume delineation followed the principles of the EORTC H10 trial and was based on pre-chemotherapy contrast-enhanced CT and FDG-PET/CT fused with the post-chemotherapy planning CT whenever feasible. The clinical target volume encompassed the initially involved nodal sites while accounting for tumor regression and respecting uninvolved normal tissues. CT-based simulation was mandatory.

Three-dimensional conformal radiotherapy and intensity-modulated radiotherapy, including volumetric techniques, were permitted. The choice of treatment technique was left to the treating radiation oncologist with the aim of achieving optimal target coverage while minimizing radiation exposure to organs at risk.

In summary, the planned treatment for LR groups 1a and 1b includes two or four chemotherapy cycles. In the case of limited relapse, INRT and nivolumab maintenance are given to both groups. In the HR group, the planned frontline treatment consists of a triple-modality regimen (four chemotherapy cycles followed by INRT and nivolumab). The extended relapse, defined as all other forms of disease relapse in the LR group or any relapse in the HR group, defines the failure of planned treatment and results in patient withdrawal from the study (Figure 2).

### 2.4. Study Procedures

The recruitment period was planned from April 2021 to December 2023. Depending on risk stratification, patients undergo 18–49 visits, including the screening and the 12 follow-up visits (one every 3 months over the 36-month follow-up period).

At screening, the following data are collected: demographic and clinical characteristics; physical examination findings; disease-specific data (histological subtype, stage, number of involved nodal areas, extranodal involvement); patient breakdown according to mEORTC criteria; laboratory test results, Eastern Cooperative Oncology Group (ECOG) performance status; and functional test results, including pregnancy testing.

At treatment visits, the following data are collected: all changes in symptoms, physical examination, laboratory results; treatments administered; and adverse events. Pregnancy is verified at each treatment visit.

Collected information data during follow-up visits include date and extension of relapse in case of treatment failure; subsequent lymphoma treatment; secondary malignancies, cardio-vascular disease, and other organ toxicity; and survival.

Patients achieving complete metabolic response (CMR) on both PET-2 and end-of-treatment PET (EoT-PET) by blinded independent central review (see below) will undergo routine follow-up: clinical and laboratory evaluations every 3 months for the first 3 years, and additionally, a cfDNA assay (every 3 months during the first year, and every 6 months during the second year). PET imaging is performed only when indicated by clinical symptoms or laboratory abnormalities. If PET suggests HL relapse, a biopsy is strongly recommended to confirm the diagnosis of disease recurrence. Patients meeting criteria for limited relapse are included in group 2 and receive “delayed” INRT followed by nivolumab 240 mg every 14 days for 1 year.

Routine clinical data will be documented at study sites and entered into a secure, web-based electronic case report form (eCRF) system (The diCELLa eCRF Clinical Trials platform) (https://dicella.com/ecrf/ assessed on 14 August 2026). Documentation is sourced from routine medical records and verified for completeness through centralized and on-site monitoring by medical managers and clinical monitors. Imaging data and cfDNA are integrated with clinical data in the eCRF.

The imaging schedule in the RAFTING trial includes baseline PET for staging, interim PET after two cycles of chemotherapy (PET-2), PET at the end of chemotherapy (EoC-PET), and PET at the end of treatment (EoT-PET). In LR group 1a patients, PET-2, EoC-PET, and EoT-PET correspond to the same scan. In LR group 1b patients, EoC-PET coincides with EoT-PET. In high-risk (HR) or relapsed patients, EoT-PET is performed after completion of nivolumab therapy. Thus, the timing of EoT-PET depends on the risk group and treatment duration.

Response is assessed using the Lugano 2014 Response Criteria [17] for chemotherapy-treated patients and LYRIC criteria for those receiving nivolumab [18].

All PET/CT examinations are performed according to the imaging governance framework established by the Cuneo Core laboratory (CoreLab) to ensure analytical validity, reproducibility, and quantitative comparability of PET-derived measurements across participating centers. Participating centers are required to use PET/CT scanners that have successfully completed the FIL Clinical Trial Qualification (CTQ) program. Scanner qualification is based on standardized phantom calibration procedures using 18F-filled cylindrical phantom reference sources, ensuring harmonization of standardized uptake value (SUV) measurements across scanners and institutions [19]. Patient preparation, radiotracer administration, uptake time, acquisition parameters, and image reconstruction are performed according to international procedural standards consistent with EANM guidelines for PET/CT imaging [20]. Compliance with protocol-defined acquisition parameters is verified through automated quality control procedures implemented within the web-based imaging platform [21].

PET images acquired at baseline, PET-2, and EoT-PET are uploaded via the platform for image exchange and review Web-Based Imaging Diagnosis by Expert Network (WIDEN) [22]. Centralized image interpretation is conducted through a blinded independent central review (BICR) performed by three experienced nuclear medicine physicians. Reviewers were blinded to clinical information and treatment allocation.

Baseline TMTV was measured independently by two central nuclear medicine reviewers on baseline PET images using semi-automated lesion segmentation software (Fiji—petctviewer.org, software version 1.53–1.54). All lymphoma-related fluorodeoxyglucose (FDG)-avid lesions were identified and segmented using a threshold corresponding to 41% of each lesion’s SUVmax [22]. If needed, lesions were edited by the operator to exclude uptake unrelated to lymphoma or to include lesions not captured by the automatic segmentation process. Physiological uptake and non-lymphomatous FDG-avid findings were excluded by expert visual review, and the metabolic volumes of all lesions were summed to derive whole-body TMTV.

Inter-reader TMTV agreement was assessed using the percentage difference between the two measurements and their mean value in relation to the prespecified 84 mL stratification threshold. When the percentage difference was <10% and the mean TMTV was between 71 and 97 mL, the mean of the two measurements was assigned as the final TMTV. When the percentage difference was <20% and the mean TMTV was either <71 mL or >97 mL, the mean value was likewise assigned. In all other cases, CoreLab reviewed the lesion segmentations and corresponding RT structures to identify the source of disagreement and then performed a consensus assessment.

PET-2 scans were independently reviewed by experienced central readers using the Deauville five-point scale. A final PET-2 classification was assigned when at least two reviewers reached concordant conclusions. If agreement was not achieved, an additional independent review was requested. The Imaging CoreLab evaluated the source of disagreement, including lesion identification, reference-organ assessment, image quality, and response categorization. Selected unresolved cases underwent consensus review [23]. The final centrally assigned PET-2 result was used for treatment allocation. PET-2 scans with Deauville scores 1–3 were classified as negative, whereas scores 4–5 were considered positive (DS 4: uptake of the reference lesion (RL) moderately higher than liver uptake; DS 5: uptake of the reference lesion higher than the liver uptake and calculated as follows: SUVmax RL > 2x SUVmax of the liver).

In patients treated with nivolumab, the final (end-of-treatment) PET/CT was scheduled approximately four weeks after the last nivolumab dose. According to the protocol, this assessment was to be interpreted using the LYRIC criteria. In the case of an indeterminate response (IR), the PET/CT scan is reviewed in consensus by the reviewers to confirm IR. PET/CT in follow-up is recommended only in the following situations: clinical suspicion of relapse (appearance of symptoms or new findings on physical examination); reappearance of cfDNA after previous negative result; a 2-log increase in cfDNA in the blood.

CfDNA is measured at baseline and after two chemotherapy cycles (PET-2) in all patients. In addition, in LR patients entering CMR, cfDNA is assessed every three months during the first year and every six months during the second year.

Peripheral blood samples (20 mL) are collected in cell-free DNA BCT tubes (Streck) and centralized at the Institute of Oncology Research (Prof. Davide Rossi’s Lab). Plasma is processed to obtain at least 30 ng of cfDNA (~5000 genome equivalents), and germline genomic DNA (gDNA) is isolated from peripheral blood mononuclear cells. Longitudinal assessment of cfDNA for genotyping and residual disease quantification is performed using the LyV4.0 CAPP-seq assay (Cancer Personalized Profiling by Deep Sequencing) (CAPP-seq) [24]. The assay targets 344 kb of genomic space, including 33 Activation-Induced Cytidine Deaminase (AID)-associated somatic hypermutation (SHM) noncoding regions [25,26,27] and coding regions of 155 recurrently mutated genes in mature B-cell tumors. A complete list of the targeted genes and genomic regions, together with the analytical validation of the assay, has been previously reported (Pirosa et al., Supplementary Material) [24].

To ensure genomic fidelity and mitigate batch biases, patient-derived cfDNA and matched gDNA were sequenced simultaneously. Sequencing was performed on the Illumina NextSeq500 platform (paired-end, 2 × 150 bp). This paired approach, combined with background-error suppression models derived from healthy cohorts, enabled the precise isolation of true somatic mutations while filtering out inherited polymorphisms and sequencing artifacts (detailed bioinformatics and laboratory methodologies are described elsewhere).

The limit of blank (LoB) was first established from the distribution of background variant allele frequencies observed in 119 post-treatment cfDNA samples from patients cured of lymphoma (blank samples). The limit of quantification (LoQ) was then calculated as LoB + 10 × standard deviation (LoB), thereby defining the lowest variant allele frequency that could be reliably quantified above background noise. Conversely, the limit of detection (LoD) for residual cfDNA was assessed by serial dilution experiments using diagnostic cfDNA samples mixed with cfDNA from healthy donors.

A critical feature of this protocol is the assay’s analytical sensitivity of 0.1%, determined through serial dilution experiments during assay validation [24]. This level of sensitivity, achieved through targeted enrichment and ultra-deep sequencing with a minimum of 2000× coverage across ≥80% of target regions, enables the reliable detection of very low-frequency tumor-derived variants. This ultra-high sensitivity is essential for accurately evaluating minimal residual disease (MRD) and detecting molecular relapse prior to radiographic or clinical progression.

Bioinformatic analysis includes alignment to the reference genome and somatic variant calling by comparison with matched gDNA. Multiple error-suppression steps are applied, including statistical filtering and comparison with background cfDNA data from healthy individuals, to ensure high specificity.

cfDNA concentration is calculated by multiplying the cfDNA concentration in plasma (pg/mL) by the mean allele fraction of patient-specific reporter mutations and dividing by 3.3 (pg per haploid genome equivalent) and is expressed on a log10 scale.

Within this study protocol, negative MRD was defined as the complete clearance of all baseline somatic alterations—including missense substitutions and short indels—to levels below the assay’s limit of quantification, as previously established during assay validation [25].

PET/CT in follow-up is recommended in the following cases: clinical suspicion of relapse (symptoms or new findings on physical examination); reappearance of cfDNA after previous negativization; a 2-log increase in cfDNA in the blood.

### 2.5. Sample Size

The sample size calculation was based on the following assumptions: the anticipated 3-year progression-free survival (PFS) rate for stage I–IIA non-bulky eHL patients with a negative PET-2, treated with CMT, is 94% [6]; the expected 3-year PFS for PET-2-negative patients with similar clinical characteristics, treated with chemotherapy alone comprising two to four courses of chemotherapy, ranges between 87% and 93%, as reported in three prospective and one retrospective clinical trials, despite variations in patient characteristics and treatment intensity [6,7,8,28].

The expected 3-year PFS for PET-2-negative patients with TMTV < 84 mL treated with chemotherapy alone is anticipated to be at least 90%. In this context, a one-sample non-inferiority design was employed to calculate the sample size required to test whether the proportion of low-risk eHL patients, treated with chemotherapy alone, alive and disease-free at 3 years, is non-inferior to that of patients treated with CMT. For the non-inferiority assessment of the primary endpoint, we assumed an expected true response proportion of 90%. The non-inferiority historical reference value was set at 95%, reflecting the historical outcome of CMT reported in trials such as RAPID and H10, with an absolute non-inferiority margin of 10 percentage points; thus, non-inferiority would be concluded if the response proportion in the study population exceeds 85%.

To define the sample size of patients to be enrolled, we used a one-sided type I error of 0.20 and type II error of 0.20 (80% power). “The one-sided α of 0.20 was prespecified because the study was designed as an exploratory Phase 2 screening trial, in which minimizing false-negative findings was prioritized over controlling false-positive findings, consistent with the recommendations of Rubinstein et al.” [29].

A total of 102 low-risk (defined by a low MTV and a negative PET-2) eHL patients should be enrolled to exclude a difference between the true proportion and the comparison value of at least 10%. Considering an expected 75% of patients with a negative PET-2 and low TMTV, and a drop-off rate of 15%, 160 patients had to be enrolled in the trial.

In the exploratory phase 2 setting, a one-sided α = 0.20 was chosen because the potential harm of a false negative result (failing to recognize an effective treatment) is considered more significant than that of a false positive result (proceeding to confirmatory testing) [29].

### 2.6. Statistical Methods Used to Compare Groups for Primary and Secondary Outcomes

The primary efficacy endpoint is 3-year progression-free survival (PFS) in the LR group, which will be evaluated for non-inferiority relative to a prespecified historical control rate. The PFS in the LR group is defined as the time from LR patient registration to the first occurrence of disease progression (limited or extended relapse) or death for any reason. The PFS distribution will be estimated using the Kaplan–Meier product-limit method, and the standard error of the 3-year survival estimate will be computed using Greenwood’s formula. For the primary hypothesis, a one-sided 80% confidence interval (CI) for the 3-year PFS rate will be constructed. Non-inferiority will be concluded if the lower bound of this one-sided 80% CI exceeds 85%, corresponding to a one-sided type I error rate (alpha) of 0.20.

The first secondary endpoint is also used for LR patients only. The 3-year freedom from second treatment failure (FF2TF) is calculated for LR who have limited relapse only and are rescued according to the protocol. It is measured from registration to the date of second relapse (during or after RT followed by nivolumab) (extended relapse) or death for any reason. FF2TF will be estimated using the Kaplan–Meier method, and the 3-year FF2TF estimate will be reported with a two-sided 95% confidence interval (CI) derived using Greenwood’s formula.

The second secondary efficacy endpoint is the 3-year PFS in the HR cohort, defined as the time from HR patient registration to the first occurrence of disease progression (extended relapse) or death for any reason. The 3-year PFS estimate will be obtained from the Kaplan–Meier curve, with a two-sided 95% CI calculated using Greenwood’s formula.

Both secondary assessments will allow us to calculate 3-year event-free survival (EFS) across the entire study cohort. The EFS is defined as the time from patient registration to the failure of the planned study treatment, which varies by study groups and includes only extended relapses or death for any reason. This is considered one of the most important study aims, reflecting the efficacy of a flexible-intensity and personalized treatment offered to different eHL prognostic groups. The 3-year EFS estimate will be obtained from the Kaplan–Meier curve, with a two-sided 95% CI calculated using Greenwood’s formula.

The third secondary endpoint is 3-year overall survival (OS) of the entire cohort of patients enrolled into the study, defined as time from patient registration to death for any reason. The OS will be estimated using the Kaplan–Meier method, with a two-sided 95% CI based on Greenwood’s standard error.

As this is a single-arm evaluation, analyses of the secondary endpoints will be descriptive and will report point estimates with corresponding measures of precision, without formal hypothesis testing against a concurrent comparator. The summary of the study outcomes is presented in Table 1.

Finally, the diagnostic performance of cfDNA for detection of an impending relapse during follow-up in LR group patients will be summarized using overall accuracy, sensitivity, specificity, negative predictive value, and positive predictive value. Each metric will be presented as a point estimate with a two-sided 95% CI computed using the Clopper–Pearson exact method.

As an intention-to-treat (ITT) approach is preferred for phase III randomized clinical trials and a per protocol (PP) approach is preferred for equivalence and non-inferiority trials, a PP analysis will be preferred for assessing the primary study endpoint of treatment efficacy in LR patients, and an ITT approach will be used as secondary [30]. For all the other study endpoints in the RAFTING study, both an ITT and PP will be used.

### 2.7. Missing Data Handling in the Analysis

In general, missing data will not be imputed for safety, demographic, or baseline characteristic variables; analyses will be based solely on observed data. For the primary endpoint and other time-to-event secondary endpoints, missing outcome information due to loss to follow-up or withdrawal of consent prior to an event will be addressed through right-censoring at the date of the last adequate disease assessment. These analyses assume that missingness is consistent with a missing at random (MAR) mechanism and that censoring is non-informative, such that the censoring process is independent of the event process conditional on the information available.

### 2.8. Methods for Any Additional Analyses

To evaluate the consistency of the treatment effect across the study population, subgroup analyses will be performed for the primary endpoint (3-year PFS) and key secondary endpoints. As this is a single-arm study, these analyses will be descriptive and exploratory. Point estimates and 95% confidence intervals will be presented for each subgroup. No formal hypothesis testing for interaction is planned. The prespecified subgroups based on baseline characteristics include:

Demographics: Age (e.g., ≤40 years vs. >40 years), sex (male vs. female);

Clinical Characteristics: Presence or absence of LNM in LR patients.

### 2.9. Toxicity and Safety Monitoring

All patients who have received at least one dose of the study treatment will be evaluable for toxicity from the time of their first drug administration. When toxicity occurs, it should be graded according to the National Cancer Institute Common Toxicity Criteria, version 5.0. Any clinically significant abnormality persisting at the end of the study will be followed up by the investigator until resolution or until reaching a clinically stable endpoint.

The safety parameters are evaluated by an external Independent Data Safety and Monitoring Committee (IDSMC) during two meetings (2 months after the first 40 patients enrolled or earlier if any emerging safety signals appear and 2 months after the next 40 patients enrolled or earlier if any emerging safety signals appear). Following each meeting, the IDMSC prepares a report and may recommend changes in the study conduction.

The interim analysis was planned after enrollment of the first 50 patients. Recruitment will be stopped if the incidence of second HL relapses among LR patients exceeds the prespecified threshold (10 ± 5%). No early stopping boundary for efficacy was planned because this exploratory non-inferiority study is intended to complete the prespecified recruitment and follow-up to estimate the primary endpoint with the planned precision.

## 3. Discussion

HL, especially in early stages, remains a model of success in oncology, with survival rates around 90% [31]. Nevertheless, patients with eHL still demonstrate heterogeneity in treatment response, ranging from an extremely favorable subgroup that can be cured with chemotherapy alone to an extremely unfavorable subgroup that does not respond to standard CMT and subsequent lines of treatment [31]. Moreover, since the turn of the millennium, several novel prognostic factors derived from functional imaging and molecular biology have emerged to guide treatment intensity and to detect minimal residual disease at the end of therapy. The RAFTING trial is the first prospective study in eHL specifically designed to address both polarized patient populations. It proposes a highly individualized treatment strategy using easily available tools aimed at maximizing efficacy while minimizing toxicity.

To stratify patients with variable prognosis, baseline TMTV, mEORTC criteria, and PET-2 results were applied. All of them were introduced based on the results of previous studies, including studies involving patients with characteristics similar to those of the RAFTING trial, often including a radiation-free treatment arm, which clearly showed that 60% of early favorable HL patients could be cured with chemotherapy alone.

Finally, data from these studies also demonstrated that approximately 30% of PET-2-negative patients who relapsed in initially involved nodes were effectively salvaged with RT alone. These observations, together with the excellent results achieved with anti-PD1 monoclonal antibodies at various treatment stages in HL provided the rationale for introducing a triple treatment in the HR group and a rescue approach with INRT and nivolumab maintenance in the LR group in case of limited relapse. This strategy may allow avoidance of the toxicity associated with HDCT and auto-HCT. The integration of delayed RT and nivolumab in patients with limited relapse in LR group ensures that even those with suboptimal initial responses have access to potentially curative interventions.

The sample size calculation was based on the proportion of patients cured with RFT in previous prospective studies. Consequently, the primary endpoint of this phase 2 prospective, single-cohort clinical trial is the 3-year PFS in the low-risk group of patients treated with chemotherapy alone. The non-inferiority design provides a rigorous framework for determining whether radiation-free chemotherapy regimens can achieve efficacy comparable to conventional CMT within a 10% margin in this subgroup. The 10% non-inferiority margin was chosen based on statistical reasoning, clinical judgment, and the feasibility of implementing the study plan, balancing the risk of reduced disease control against the potential long-term benefit of omitting radiotherapy. A narrower margin, such as 5%, would have required a substantially larger sample size, approaching that of a confirmatory trial. Therefore, the present study is intended to exclude clearly unacceptable loss of efficacy and to inform the design of a subsequent phase 3 trial using a more stringent margin. We also referred to prior Hodgkin lymphoma trials that used a 7–10% non-inferiority margin to define clinically meaningful differences in efficacy (RAPID trial—7%, EORTC/LYSA/FIL H10—10%) [6,7].

Equally important is the main secondary endpoint, the 3-year EFS in all groups of patients, which will allow estimation of the proportion of patients who fail the planned treatment. Thus, the limited relapse in LR patients treated with delayed INRT and nivolumab is not considered as an event. The events defining the true failures of planned treatment include only extended relapse at any time or any relapse including limited relapse after completing the triple treatment.

The RAFTING trial is also the first prospective study to evaluate the role of cfDNA in eHL. By investigating cfDNA as a predictor of impending relapse, the trial aligns with the increasing interest in liquid biopsies as a non-invasive monitoring tool. This approach has the potential to enable earlier interventions in patients at risk of relapse and may ultimately improve long-term outcomes. Subsequent studies could refine cfDNA thresholds, assess cost-effectiveness, and evaluate the applicability of this approach in other lymphoma subsets.

Nonetheless, the protocol faces important challenges. The complexity of the study design may lead to deviations in treatment or procedures. Another very important limitation is the single-arm design and reliance on comparison with a historical control. Such comparisons may be influenced by temporal changes in chemotherapy application, supportive care, imaging and RT techniques, and other aspects of clinical practice. However, the standard first-line treatment of eHL, based on chemotherapy with PET-adapted strategies, has remained largely unchanged over the past two decades, with stable results, including a 3-year PFS of 95%. Improvements in supportive care are unlikely to have substantially influenced PFS. The influence of improvements in imaging and RT techniques cannot be ruled out in a single-arm study with a historical comparator. We selected the RAPID trial as the principal historical benchmark because it represents a well-characterized PET-adapted cohort with similar, although not identical, eligibility criteria and RT technique (INRT). In addition, response assessment in the comparator studies was performed using blinded independent central review, reducing variability in PET interpretation, as in the RAFTING study. Nevertheless, advances in PET/CT acquisition, reconstruction algorithms, scanner performance, and staging procedures over time may have influenced patient classification and outcome assessment. Therefore, comparisons with historical controls should be interpreted with caution, and our findings require confirmation in prospective randomized or contemporaneous comparative studies. Another limitation of the study is related to future implementation of the study results, related to potential variability in imaging interpretation in the real-world setting, particularly regarding the assessment of PET-2 and baseline TMTV. The central review process has been implemented to minimize this risk in the study. Moreover, the BICR of a given test is needed when the study endpoint depends directly on this test [23]. The real-world application of these criteria may lead to inconsistencies across treatment centers. Similarly, the use of cfDNA as a biomarker, although highly promising, remains exploratory, and definitive conclusions may be limited by inherent variability in assay sensitivity and specificity. Additional challenges relate to the details of the treatment protocol. The initially planned schedule of 24 nivolumab doses may prove difficult to achieve in all patients, and available data suggest that shorter treatment courses might still provide adequate disease control in this setting.

Future validation against independent contemporary cohorts would provide additional reassurance regarding the reproducibility and generalizability of the present findings. Sensitivity analyses exploring the impact of alternative comparator populations or analytical assumptions could further evaluate the robustness of the results. However, these analyses are beyond the scope of the current exploratory phase 2 study.

We would like to emphasize that any positive result of the study will reflect the whole study algorithm, not the separate checkpoint of treatment blocks.

The recruitment of patients was completed in October 2023, and the target sample size was achieved. All patients finished the planned per-protocol treatment. Clinical follow-up is ongoing according to the study protocol.

To conclude, if the RAFTING trial confirms the efficacy of RFT in low-risk patients defined with new proposed tools, it will provide strong arguments for revising current treatment guidelines. Incorporating TMTV and mEORTC criteria together with PET-2 into risk stratification could become a cornerstone of HL management, supporting a more individualized approach with reduced long-term toxicity. If successful, the study findings will have significant implications for the management of HL, particularly by reducing the incidence of long-term complications such as secondary malignancies and cardiovascular disease.

All information generated by the RAFTING study is owned by the study sponsor, the Medical University of Gdansk. The latter will have the discretion of committing to the Principal Coordinating Investigator or his delegates to disseminate the results of the study to the medical community. All information and results derived from this trial will be considered confidential, at least until such time as appropriate analysis and verification have been concluded by the sponsor, the coordinating investigators, and the trial statistician.

The results of the study will be disseminated through publication in peer-reviewed scientific journals and presentation at national and international scientific conferences. The findings will be reported in accordance with relevant reporting guidelines and will be published irrespective of the nature or direction of the results.

## Figures and Tables

**Table 1:** The summary of study outcomes.

End-Point	Type	Group of Patients	Events
3-year progression-free survival (PFS) in low-risk group	Primary	Low-risk	Any progression (limited and extended relapse—whatever occurs first); death of any reason
3-year freedom from second treatment failure (FF2TF)	Secondary	Low-risk with limited relapse rescued in protocol (radiation therapy + nivolumab)	Progression during or after rescue treatment (extended relapse); death of any reason
3-year progression-free survival (PFS) in high-risk group	Secondary	High-risk	Extended relapse; death of any reason
3-year event-free survival (EFS)	Secondary	Entire cohort	Extended relapse (failure of planned per-protocol treatment—varies depending on stratification); death of any reason
3-year overall survival (OS)	Secondary	Entire cohort	Death of any reason

## Data Availability

The dataset presented in this article is not readily available because is the part of an ongoing study. Requests to access the datasets should be directed to the corresponding author.
